# Brown Norway ovalbumin model: temporal profile of cytokines

**DOI:** 10.1186/1476-9255-10-S1-P9

**Published:** 2013-08-14

**Authors:** Janice D Woodhouse, Alan Wilhelm, Michael Caniga, Malgorzata Gil, Jie Zhang-Hoover, Robbie McLeod, Lily Y Moy, Milenko Cicmil

**Affiliations:** 1 Merck Research Laboratories, Boston, MA 02115, USA

## Background

The ovalbumin (OVA) sensitized and challenged Brown Norway (BN) rat model is a practical PD model often used to determine the impact of drug treatment on late phase lung inflammation. However, the sole measurement of bronchoalveolar lavage fluid (BALF) inflammatory cells does not always correlate into human efficacy for respiratory diseases such as asthma. Added value to this rodent model may be derived by a deeper understanding of the relationship between disease-related cytokine secretion and inflammation.

## Materials and methods

Animal sensitizations, challenges, and BALF collections were performed as described previously [1]. Inflammatory cell counts and cytokine temporal profile was investigated in BALF samples collected from animals at 6, 24, 48, and 72 hours post whole-body OVA challenge. Effect of orally dosed betamethasone on the temporal profile of inflammatory cells and cytokines was also investigated.

## Results

Differential cell counts from BALF showed an increase in neutrophil levels at 6h, reaching maximum at 24h, in OVA challenged animals. The influx of eosinophils in BALF continues to increase with time, reaching peak at 72h. Baseline levels for a panel of cytokines (including IL-13 and IL-5) were also established with most reaching maximal levels at 24h. A significant inhibition of neutrophil levels was seen in the betamethasone group at 6h (71%) and 24h (67%) along with inhibition of IL-13 (90 %) at 24h. Betamethasone was also able to significantly inhibit eosinophilia and IL-13 at 48h time point.

## Conclusions

We were able to confirm the temporal profile of cytokine secretion in BALF in this model. In addition, we have shown that betamethasone inhibited both cytokines and cell infiltration in a dose dependent manner. The cytokine endpoint in conjunction with cell influx brings added value to the standard OVA challenged Brown Norway rat model.
